# Quality Management Outweighs Pandemic: Retrospective Analysis Shows Improved Quality of Care for *Staphylococcus aureus* Bacteremia Despite SARS-CoV-2

**DOI:** 10.3390/diseases13040104

**Published:** 2025-03-30

**Authors:** Lena Jakoby, Ernst Molitor, Nico T. Mutters, Ruth Weppler, Dominic Rauschning, Manuel Döhla

**Affiliations:** 1 Institute for Hygiene and Public Health, Medical Faculty, University of Bonn, 53127 Bonn, Germany; 2 Department of Microbiology and Hospital Hygiene, Bundeswehr Central Hospital Koblenz, 56072 Koblenz, Germany; 3 Institute of Medical Microbiology, Immunology and Parasitology, Medical Faculty, University of Bonn, 53127 Bonn, Germany; 4 Division of Infectious Diseases, Department IB of Internal Medicine, Bundeswehr Central Hospital Koblenz, 56072 Koblenz, Germany; 5 Division of Infectious Diseases, Department I of Internal Medicine, University of Cologne, 50923 Köln, Germany

**Keywords:** staphylococcal infections, *Staphylococcus aureus*, bacteremia, SARS-CoV-2, quality of health care, antimicrobial stewardship

## Abstract

Background: *Staphylococcus aureus* bacteremia (SAB) is of great clinical relevance, as it is the most common type of bacteremia. Several studies show that the quality of care and thus the outcome can be positively influenced by the involvement of infectious disease specialists and structured programs like Antimicrobial Stewardship (AMS). In 2020, the SARS-CoV-2 pandemic occurred, which dominated the healthcare system and global events during this time. At the same time, a standard operational procedure (SOP) for SAB quality management (SABQM) was introduced in a German maximum-care hospital with 500 beds. Additionally, voluntary AMS team consultations were introduced in June 2021. This work addresses whether the introduction of SABQM has led to an improvement in the quality of care for SAB, despite the possible negative influences of the pandemic. Methods: Retrospective statistical analyses were conducted on all 145 cases coded as SAB at this hospital during the “pre-pandemic” period (2017 to 2019, 75 cases) and the pandemic period (2020 to 2022, 70 cases). Population parameters and quality management parameters were extracted from the clinical patient documentation. In a first analysis, the SARS-CoV-2 status served as a discriminatory parameter to determine its influence on the quality of care within the “pandemic period”. In a second analysis, the period served as a discriminatory parameter to determine its influence on the quality of care. In a third analysis, the use of AMS team consultation served as a discriminatory parameter to determine its influence on the quality of care in a subgroup of 42 cases from June 2021 to 2022. Results: The SARS-CoV-2 status had no influence on the population parameters or the quality management parameters. Between both analyzed periods, there was an improvement in the quality management parameters, with statistically significant higher rates of follow-up blood cultures, transthoracic echocardiography and adequate antibiotic therapy. AMS team consultation led to a relevant, but not statistically significant improvement in the quality management indicators. Conclusions: An SOP for SABQM leads to an improvement in the quality of care, even under the possible negative influences of a pandemic. AMS team consultations further strengthen this positive influence, even if this is not statistically significant due to the small number of cases in the subgroup analyzed.

## 1. Introduction

*Staphylococcus aureus*, a gram-positive bacterium, is one of the most common causes of bacteremia [1,2]. *S. aureus* bacteremia (SAB) can be classified according to the pathogen’s susceptibility, such as methicillin-sensitive (MSSA) or methicillin-resistant (MRSA) [1,2,3,4]. Another classification method distinguishes between uncomplicated and complicated SAB [5]. Complicated SAB refers to cases where fever does not reduce after 48–72 h of antibiotic therapy, infectious foci cannot be eradicated, septic metastases occur or endovascular prostheses have been placed, or infectious endocarditis is present. The absence of these factors characterizes an uncomplicated SAB.

With an incidence of 9.3 to 65 per 100,000 people per year [4] and a high mortality rate of up to 30% [3], SAB is of great clinical relevance. That is why structured measures for infection prevention and control are particularly important. Intravenous catheters are considered to be the main route of transmission of *S. aureus* [3]. For infection prevention, the focus is on avoiding transmissions through “catheter bundles,” which include strict hand hygiene, aseptic techniques during catheter placement and daily evaluation of the necessity for indwelling catheters [6]. For infection control, several measures are described that can lead to a better outcome for patients [5]: follow-up blood cultures after every 48 h, echocardiography to rule out endocarditis, identification of possible metastases via imaging, source control (e.g., removing catheters or explanting devices), consultation of infectious disease (ID) specialists, selection of the best antibiotics based on microbiological resistance testing, and an adequate duration of therapy.

The Bundeswehr Central Hospital in Koblenz, a hospital of maximum care with 500 beds, introduced a standard operational procedure (SOP) for SAB quality management at the beginning of 2020 based on a recommendation from 2019 [7]. At the same time, the hospital was getting involved in the fight against the SARS-CoV-2 pandemic [8]. Studies are inconclusive whether [9] or not [10] the SARS-CoV-2 pandemic has had an impact on the quality of treatment for SAB.

This study therefore aims to examine how this constellation affected the management of SAB. On the one hand, the introduction of an SOP for SAB quality management is expected to improve SAB management, while on the other hand, negative effects resulting from the SARS-CoV-2 pandemic could prevail. The hypothesis was that the positive effects of an SOP for SAB quality management outweigh any negative effects of the SARS-CoV-2 pandemic.

## 2. Materials and Methods

A retrospective secondary data analysis was conducted on all cases per year coded as SAB at the Bundeswehr Central Hospital Koblenz during the “pre-pandemic period” from 2017 to 2019 and during the “pandemic period” from 2020 to 2022. Misclassified cases, in which there was no laboratory evidence of *S. aureus*, were excluded (“pre-pandemic period”: 2 cases; “pandemic period”: 1 case).

### 2.1. Population Parameters

The following population parameters were recorded: age [in years], sex [male/female], duration of hospital stay [in days], MRSA [yes/no], Charlson Comorbidity Index (CCI) [11] [0 to 34], clinical outcome [dead/healed/discharged under therapy] and source of infection, if known [catheter-related/skin and soft tissue/lung/bone and joint/infectious endocarditis/urinary tract/meningitis/unidentified]. For the pandemic period, the SARS-CoV-2 status [positive/negative] was also recorded. The vaccination status with regard to SARS-CoV-2 was not recorded for the patients, as it was not systematically documented.

### 2.2. Quality Management Parameters

All quality management parameters were obtained from the patient files applying for each a four-eyes principle by two of the authors (L.J. and an ID specialist, either D.R. or M.D.).

The following six quality management parameters from the introduced SOP for SAB quality management were obtained [7]:

1. Follow-up blood cultures: This parameter was considered to be met if follow-up blood cultures were taken every 48 to 96 h from the first positive blood culture until sterility was achieved.
2. Echocardiography: This parameter was considered to be met if at least one transthoracic echocardiography (TTE) or transthoracic echocardiography (TEE) was performed. If only a TTE was performed, it was not determined whether an additional TEE would have been necessary or useful.
3. Focus/metastasis search: This parameter was considered to be met if either a focus/metastasis was documented or, despite an unidentifiable focus at the clinic, further examinations (e.g., ultrasound, CT, MRI) were documented that were based on previous findings.
4. Focus/metastasis control: This parameter was considered to be met if removal of catheters or devices or another focus/metastasis sanitation was documented.
5. Adequate antibiotic therapy: This parameter was considered to be met if treatment was started early and with a suitable antibiotic (generally for MSSA: flucloxacillin or cefazolin, for MRSA: vancomycin or daptomycin, or if necessary other agents or combinations if effective and comprehensibly justified) and the duration of therapy was sufficiently long (uncomplicated SAB: at least 14 days after the first negative blood culture, complicated SAB: at least 4 weeks after the first negative blood culture, possibly longer for artificial valves or for other reasons, if comprehensibly justified).
6. Antimicrobial Stewardship (AMS) team consultation: An AMS team consisting of an ID specialist, a microbiologist, a clinical pharmacist and a hygienist has been available since June 2021 [12]. From that point on, the parameter was taken into account and considered fulfilled if an AMS consultation was requested in the hospital information system.

The “Quality of Care Indicator” (QCI) was calculated as reported in other studies that addressed SAB quality management [13,14]. QCI is the sum of met parameters 1, 2, 4 and 5 and has a range from zero to four.

### 2.3. Data Analysis

To test the hypothesis that the positive effects of an SOP outweigh any negative effects of the SARS-CoV-2 pandemic, three partial analyses were carried out:

Firstly, within the “pandemic period”, SARS-CoV-2-negative cases were compared with SARS-CoV-2-positive cases. This was done to control for a negative effect of the pandemic at the micro (patients with SARS-CoV-2 and SAB could be sicker or older and therefore more likely to have negative outcomes) and meso level (SARS-CoV-2-positive patients could receive less, poor or wrong diagnostics and therapy).

Secondly, cases in the “pre-pandemic period” were compared with those in the “pandemic period” to weigh up a possible negative effect of the pandemic at the macro level (complete focus on SARS-CoV-2, ignoring all other clinical pictures, especially in infectious diseases) against the expected positive effect of an SOP.

Thirdly, cases within the “pandemic period” were compared from June 2021 in terms of the use of the AMS team consultation to determine the effect of this measure within the patient group.

All statistical tests were performed via STATA IC 15.1 (Stata Corp, Texas, USA). Binary variables were reported as absolute number and as relative percentage; comparisons between groups were conducted using 2-sided Fisher’s exact test at an alpha level of 0.05. Continuous variables were reported as median and interquartile range; comparisons between groups were conducted using 2-sided Mann–Whitney U test at an alpha level of 0.05.

## 3. Results

A total of 145 cases could be included in the secondary data analysis, 75 of them in the “pre-pandemic period” and 70 in the “pandemic period”. Within the “pandemic period”, 59 included cases were SARS-CoV-2-negative, and 11 included cases were SARS-CoV-2-positive.

### 3.1. Comparison of SARS-CoV-2-Negative and SARS-CoV-2-Positive Cases Within the “Pandemic Period”

SARS-CoV-2-positive cases had a significantly higher CCI than SARS-CoV-2-negative cases (5 and 9, respectively, *p* = 0.026). All other population and quality management parameters recorded were statistically unremarkable and, even without statistical analysis, showed no clear tendency, so that based on the data, it was assumed that SARS-CoV-2-negative and SARS-CoV-2-positive cases did not differ in terms of population parameters and quality management parameters (Table 1). Therefore, all cases were included in further analyses, regardless of their SARS-CoV-2 status.

### 3.2. Comparison of Cases Between the “Pre-Pandemic Period” and the “Pandemic Period”

**Figure 1:**
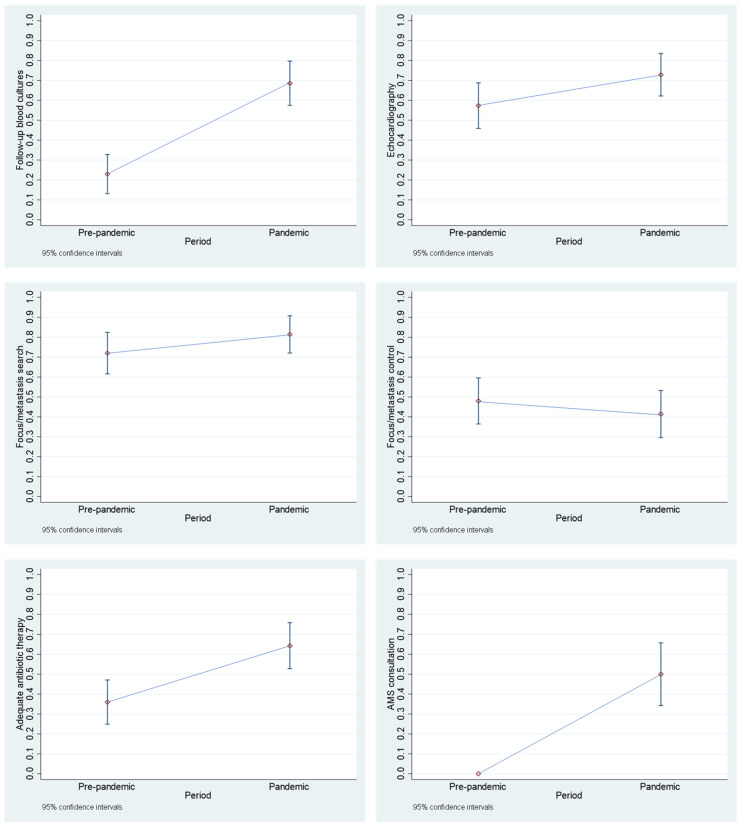
Change in quality management indicators between the “pre-pandemic period” and the “pandemic period”. The respective point estimate and the 95% confidence interval are given.

There were no differences in the population parameters between the two periods. Almost all quality management parameters improved in trend (Figure 1), except for a statistically not significant early focus/metastasis removal (41.4% vs. 48.0%, *p* = 0.504). However, the rates of follow-up blood cultures (68.6% vs. 22.6%, *p* = 0.000), TEE (58.6% vs. 38.7%, *p* = 0.020), adequate antibiotic therapy (64.3% vs. 36.0%, *p* = 0.001) and QCI (2 vs. 1, *p* = 0.000) were statistically significant. More details are given in Table S1.

### 3.3. Comparison of Cases with AMS Team Consultation and Cases Without AMS Team Consultation After June 2021 Within the “Pandemic Period”

**Figure 2:**
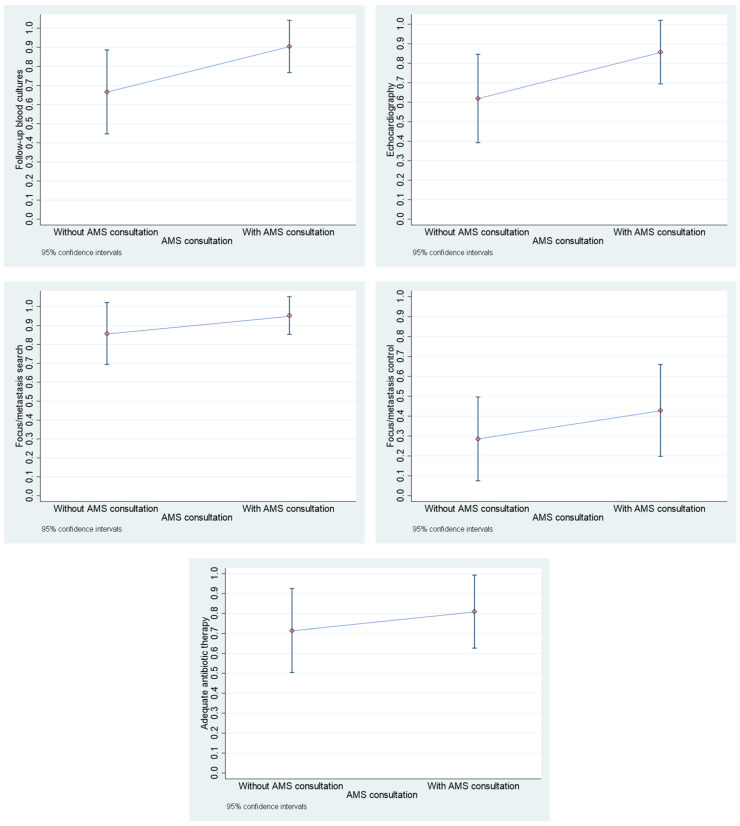
Change in quality management indicators parameters for the “pandemic period”, divided according to AMS consultation status. The respective point estimate and the 95% confidence interval are given.

There were no significant differences between the groups with and without AMS team consultation (Figure 2). Nevertheless, there is a general tendency in the quality management indicators to show a relevant improvement, albeit without statistical significance. Details are given in Table S2.

## 4. Discussion

### 4.1. Principal Findings

Three main results can be summarized:

Firstly, no relevant difference in population parameters or quality management parameters within the “pandemic period” could be detected in the examined cohort between SARS-CoV-2-negative and SARS-CoV-2-positive cases except the higher CCI of the SARS-CoV-2-positive patients.

Secondly, certain improvements within the quality management parameters could be seen between the two observation periods, the “pre-pandemic period” and the “pandemic period”, although the population parameters remained the same. This shows that the introduction of comprehensive AMS measures leads to better management of SAB even under pandemic conditions.

Thirdly, a positive effect of AMS team consultations on all quality management parameters can be assumed, even if this cannot be statistically proven in the present study due to the small number of cases and two-sided statistical testing.

### 4.2. Strengths and Limitations

One of the major strengths of this study is the unique framework conditions. The simultaneous introduction of an SOP for SAB quality management and the beginning of a major pandemic with drastic countermeasures cannot be planned in any way but is purely a product of chance.

Another strength is that all cases of SAB over a long period of six years could be included in the analysis without exception, so there is no sampling. This helps to rule out the possibility of statistical distortion of the data due to a poorly drawn sample.

A significant limitation is the relatively small number of cases included, which only allows for a descriptive analysis. In particular, the two subgroup analyses conducted according to SARS-CoV-2 status and according to AMS team consultation are clearly underpowered, so that the effects that may be present cannot be statistically verified. The findings of this study are therefore only indicative and must be critically placed in the context of the wider literature.

Another limitation is the method of retrospective evaluation, which in this study had to operate with paper case files. It is possible that the measures carried out were not documented or could not be found within the case files during the data collection, so that the reported quality indicators could be too low. This weakness in the study design was counteracted by using the four-eyes principle but cannot be completely ruled out. However, there is no evidence that this documentation bias is different in the subgroups studied.

### 4.3. Pandemic’s Negative Impact on the Mortality

This study aimed to examine how, on the one hand, the introduction of an SOP for SAB quality management, on the other hand, possible negative effects resulting from the SARS-CoV-2 pandemic affected the management of SAB.

The relevant end point of all efforts to improve SAB quality management is survival as the desired outcome. Mortality is reported in various studies to be between 20 and 30% [3,15]. The positive influence of measure bundles that optimize quality management parameters and improve outcomes is considered proven [9,14,16,17]. However, even a good SAB quality management system can be associated with high mortality, even independently of a pandemic [18]. Arientová et al., for example, report that after the introduction of an SAB quality management system in 2017/2018, mortality fell from 27% in 2016 to 6% in 2019 [9,15]. But they also report an increase in mortality during the pandemic back to 23% [9]. Lorenzo-Hernández et al. report an increase in 30-day mortality from 23.3% to 35% in the pandemic, with a mortality rate of 70.6% for SARS-CoV-2 co-infection [19]. Falces-Romero et al. report 30-day mortality rates of around 20% in patients without SARS-CoV-2 co-infection and even around 40% in patients with SARS-CoV-2 co-infection [20]. However, they do not provide any information on mortality in the “pre-pandemic period”. Abdollahi et al. report a mortality rate of more than 40% during the pandemic [21]. A negative effect of the pandemic on the outcome of the SAB is therefore obvious and probably led to the fact that mortality in our study did not decrease despite the introduction of SAB quality management.

The pandemic’s negative impact on mortality could occur at the micro, meso or macro level. At the micro level, a negative effect could be seen due to the late presentation of sick patients and the resulting delay in diagnosis and therapy. In the present study, SARS-CoV-2-positive patients had a significantly higher CCI (9 vs. 5, *p* = 0.026) and higher mortality compared to SARS-CoV-2-negative patients (54.6% vs. 28.6%). Patients’ fear of seeking medical treatment during the SARS-CoV-2 pandemic has been described in several ways [22,23,24]. This fear was not unjustified, because a SARS-CoV-2 infection can be considered a risk factor for a bacterial superinfection, including with *S. aureus* [20,25,26]. However, for patients with SAB, delayed presentation precludes the use of the “hit hard and early” strategy [27], leading to higher mortality.

At the meso level, poorer treatment of patients with and without SARS-CoV-2 is suspected. Patients with SARS-CoV-2 were and are still being isolated in single rooms in hospitals [28,29], which in itself has negative physical and psychological effects [30]. However, isolated patients fall generally short in diagnostics and treatment, whether because of staff fears of infection or because of process organization [30,31,32]. This may lead to patients with SAB and suspected or confirmed SARS-CoV-2 infection being diagnosed later or less accurately and treated sub-optimally. Consequently, a negative outcome seems to be more likely.

At the macro level, a societal and media focus on the SARS-CoV-2 pandemic and, in particular, on the occurrence of new cases could be observed [33]. This “agenda-setting effect” [33] could lead to misdiagnosis with negative outcomes [34]. But even if patients have a SARS-CoV-2 infection, they may have a bacterial superinfection, such as pneumonia caused by *S. aureus*, which can serve as a focus for SAB [26,35]. However, due to the focus of society and the media on SARS-CoV-2, further examinations may not have been carried out after the diagnosis of “SARS-CoV-2 infection” and such superinfections may have been overlooked, which can also lead to a negative outcome.

In May 2023, the WHO declared an end to the public health emergency regarding SARS-CoV-2. A future follow-up investigation to this study for the years 2023 to 2025 should show whether mortality decreases in a post-pandemic period. The improvements shown in almost all quality management parameters suggest a positive result here. In particular, the AMS team consultation could not yet be fully effective, as it was only introduced in mid-2021 and just 21 patients with consultations were included in the present study. Zeidler et al. [12] analyzed all AMS team consultations at the Bundeswehr Central Hospital Koblenz from mid-2021 to the end of 2023. They could show an increase in AMS team consultations from 28 in the second quarter of 2021 to 169 in the fourth quarter of 2023, whereby 3/4 of all AMS team consultations were requested by peripheral wards. The positive correlation between AMS team consultations—especially by ID specialists—and improved AMS in general and improved SAB quality management in particular is well documented [16,36,37,38]. And despite the small number of cases, this study already shows a tendency towards an improvement in the SAB quality management parameters. This is understandable, because the involvement of ID specialists enables expert advice and control of the measures implemented, which leads to significantly improved procedural safety, especially for physicians with little experience in dealing with ID.

### 4.4. Implications for Policy, Practice and Research

Based on the data reported in this study and the high clinical relevance of SAB, a mandatory consultation by the AMS team was introduced at the Bundeswehr Central Hospital in Koblenz in 2023. Since then, every patient in whom *S. aureus* is microbiologically detected in a blood culture is reported to the AMS team by the microbiological laboratory. The AMS team then contacts the treating physician independently and provides its expertise through personal counselling and the provision of checklists. The implementation of the defined measures is regularly monitored by the AMS team to ensure that patients with SAB receive the best possible care.

Given the great medical importance of the syndrome of SAB and the high costs for the patient and/or healthcare system associated with the long treatment, hospitals should specifically promote the training of ID specialists and, depending on their size, establish and operate appropriate ID departments.

## 5. Conclusions

The positive effects of the introduction of SAB quality management outweigh the possible negative effects of the SARS-CoV-2 pandemic regarding the quality of care. Especially when the health system is focused on other conditions, SAB quality management ensures that the diagnosis and treatment of this complex syndrome is not neglected.

## Figures and Tables

**Table 1:** Basic and quality management parameters for the “pandemic period”, divided according to SARS-CoV-2 status. N: number; DH: duration of hospital stay; MRSA: methicillin-resistant *Staphylococcus aureus*; CCI: Charlson Comorbidity Index; MSSA: methicillin-sensible *Staphylococcus aureus*; TTE: trans-thoracic echocardiographic; TEE: trans-esophageal echocardiographic; AMS: antimicrobial stewardship; QCI: quality of care index.

Discrimination Parameter			
SARS-CoV-2	Negative	Positive	
Population Parameters			*p*-Value
N	59	11	
Age (Median [IQR])	66 [58–79]	77 [52–85]	0.518
Male (n [%])	36 [61.0%]	7 [63.6%]	1.000
DH (Median [IQR])	20 [13–31]	12 [7–21]	0.088
MRSA (n [%])	3 [5.1%]	0 [0.0%]	1.000
CCI (Median [IQR])	5 [3–8]	9 [4–11]	0.026 *
Mortality (n/N [%])	16/56 [28.6%]	6 [54.6%]	0.157
MSSA (n/N [%])	16/16 [100.0%]	6/6 [100.0%]	
MRSA (n/N [%])	0/16 [0.0%]	0/6 [0.0%]	
Source of infection (n [%])			
Catheter-related (n [%])	8 [13.6%]	0 [0.0%]	0.333
Skin and soft tissue (n [%])	8 [13.6%]	1 [9.1%]	
Lung (n [%])	12 [20.3%]	3 [27.3%]	
Bone and joint (n [%])	11 [18.6%]	1 [9.1%]	
Infectious endocarditis (n [%])	7 [11.9%]	0 [0.0%]	
Urinary tract (n [%])	3 [5.1%]	1 [9.1%]	
Meningitis (n [%])	0 [0.0%]	0 [0.0%]	
Unidentified (n [%])	10 [17.0%]	5 [45.4%]	
**Quality management parameters**			*p*-value
Follow-up blood cultures (n [%])	40 [67.8%]	8 [72.7%]	1.000
Echocardiography (n [%])	42 [71.2%]	9 [81.8%]	0.715
TTE (n [%])	26 [44.1%]	3 [27.3%]	0.342
TEE (n [%])	33 [55.9%]	8 [72.7%]	0.342
Focus/metastasis search (n [%])	48 [81.4%]	9 [81.8%]	1.000
Focus/metastasis control (n [%])	27 [45.8%]	2 [18.2%]	0.108
Adequate antibiotic therapy (n [%])	37 [62.7%]	8 [72.7%]	0.735
AMS consultation (n [%])	19 [32.2%]	2 [18.2%]	0.485
QCI (Median [IQR])	2 [2–3]	3 [2–3]	0.839

* indicates a statistically significant difference.

## Data Availability

The original contributions presented in this study are included in the article/Supplementary Materials. Further inquiries can be directed to the corresponding author(s).

## Supplementary Materials

The following supporting information can be downloaded at: https://www.mdpi.com/article/10.3390/diseases13040104/s1, Table S1: Basic and quality management parameters during the “pre-pandemic period” and the “pandemic period”; Table S2: Basic and quality management parameters for the “pandemic period”, divided according to AMS consultation status.

## Abbreviations

The following abbreviations are used in this manuscript:

AMS	Antimicrobial stewardship
CCI	Charlson Comorbidity Index
DH	Duration of hospital stay
ID	Infectious diseases
MRSA	Methicillin-resistant *Staphylococcus aureus*
MSSA	Methicillin-sensitive *Staphylococcus aureus*
QCI	Quality of care index
SAB	*Staphylococcus aureus* bacteremia
SARS-CoV-2	Severe acute respiratory syndrome-corona virus-2
SOP	Standard operational procedure
TEE	Trans-esophageal echocardiography
TTE	Trans-thoracal echocardiography
WHO	World Health Organization
