# Supplementary material for: Quality Management Outweighs Pandemic: Retrospective Analysis Shows Improved Quality of Care for Staphylococcus aureus Bacteremia Despite SARS-CoV-2
Source: Diseases. 2025 Mar 30;13(4):104. doi: 10.3390/diseases13040104 (PMC12025857; doi:10.3390/diseases13040104)
Supplement: Supplementary file 1 [file diseases-13-00104-s001.zip › diseases-3517442-supplementary.pdf]

**Table S1.** Basic and quality management parameters during the "pre-pandemic period" and the "pandemic period". N: Number DH: Duration of hospital stay MRSA: Methicillin-resistant *S. aureus* CCI: Charlson Comorbidity Index MSSA: Methicillin-sensible *S. aureus* TTE: Trans-thoracic echocardiographic TEE: Trans-esophageal echocardiographic AMS: Antimicrobial stewardship QCI: Quality of care index.

| **Discrimination parameter** | | | |
|---|---|---|---|
| **Period** | **Pre-pandemic** | **Pandemic** | |
| **Years** | **2017 - 2019** | **2020 – 2022** | |
| **Population parameters** | | | **p-value** |
| N | 75 | 70 | |
| SARS-CoV-2 (n [%]) | n.a. | 11 [15.7 %] | n.a. |
| Age (Median [IQR]) | 65 [55 – 79] | 66.5 [58 – 80] | 0.519 |
| Male (n [%]) | 51 [68.0 %] | 43 [61.43] | 0.487 |
| DH (Median [IQR]) | 17 [12 – 30] | 18 [12 – 28] | 0.878 |
| MRSA (n [%]) | 6 [8.0 %] | 3 [4.3 %] | 0.496 |
| CCI (Median [IQR]) | 7 [4 – 8] | 6.5 [3 – 8] | 0.652 |
| Mortality (n/N [%]) | 21 / 71 [29.6 %] | 22 / 67 [32.8 %] | 0.716 |
| MSSA (n/N [%]) | 19 / 21 [90.5 %] | 22 / 22 [100.0 %] | 0.574 |
| MRSA (n/N [%]) | 2 / 21 [9.5 %] | 0 / 22 [0.0 %] | 0.500 |
| Source of infection (n [%]) | | | |
| Catheter-related (n [%]) | 12 [16.0 %] | 8 [11.4 %] | |
| Skin and soft tissue (n [%]) | 15 [20.0 %] | 9 [12.9 %] | |
| Lung (n [%]) | 15 [20.0 %] | 15 [21.4 %] | |
| Bone and joint (n [%]) | 6 [8.0 %] | 12 [17.1 %] | 0.653 |
| Infectious endocarditis (n [%]) | 8 [10.7 %] | 7 [10.0 %] | |
| Urinary tract (n [%]) | 4 [5.3 %] | 4 [5.71 %] | |
| Meningitis (n [%]) | 1 [1.3 %] | 0 [0.0 %] | |
| Unidentified (n [%]) | 14 [18.7 %] | 15 [21.4 %] | |
| **Quality management parameters** | | | p-value |
| Follow-up blood cultures (n [%]) | 17 [22.6 %] | 48 [68.6 %] | 0.000 * |
| Echocardiography (n [%]) | 43 [57.3 %] | 51 [72.9 %] | 0.057 |
| TTE (n [%]) | 26 [34.7 %] | 29 [41.4 %] | 0.252 |
| TEE (n [%]) | 29 [38.7 %] | 41 [58.6 %] | 0.020 * |
| Focus/metastasis search (n [%]) | 54 [72.0 %] | 57 [81.4 %] | 0.239 |
| Focus/metastasis control (n [%]) | 36 [48.0 %] | 29 [41.4 %] | 0.504 |
| Adequate antibiotic therapy (n [%]) | 27 [36.0 %] | 45 [64.3 %] | 0.001 * |
| AMS consultation (n [%]) | n.a. | 21 / 42 [50.0 %] | n.a. |
| QCI (Median [IQR]) | 1 [1 – 2] | 2 [2 – 3] | 0.000 * |

**Table S2.** Basic and quality management parameters for the "pandemic period", divided according to AMS consultation status. N: Number DH: Duration of hospital stay MRSA: Methicillin-resistant *S. aureus* CCI: Charlson Comorbidity Index MSSA: Methicillin-sensible *S. aureus* TTE: Trans-thoracic echocardiographic TEE: Trans-esophageal echocardiographic AMS: Antimicrobial stewardship QCI: Quality of care index.

| **Discrimination parameter** | | | |
|---|---|---|---|
| **AMS consultation status** | **Without AMS consultation** | **With AMS consultation** | |
| **Population parameters** | | | **p-value** |
| N | 21 | 21 | |
| SARS-CoV-2 (n [%]) | 3 [14.3 %] | 2 [9.5 %] | 0.500 |
| Age (Median [IQR]) | 66 [58 – 81] | 78 [60-84] | 0.125 |
| Male (n [%]) | 13 [61.9 %] | 14 [66.7 %] | 1.000 |
| DH (Median [IQR]) | 16 [7 – 28] | 21 [15 – 36] | 0.134 |
| MRSA (n [%]) | 0 [0.0 %] | 3 [14.3 %] | 0.232 |
| CCI (Median [IQR]) | 7 [3 – 8] | 7 [5 – 9] | 0.316 |
| Mortality (n/N [%]) | 8 [40.0 %] | 7 [33.3 %] | 0.751 |
| MSSA (n/N [%]) | 8 / 8 [100.0 %] | 7 / 7 [100.0 %] | |
| MRSA (n/N [%]) | 0 / 8 [0.0 %] | 0 / 7 [0.0 %] | |
| Source of infection (n [%]) | | | |
| Catheter-related (n [%]) | 1 [4.8 %] | 2 [9.5 %] | |
| Skin and soft tissue (n [%]) | 3 [14.3 %] | 4 [19.0 %] | |
| Lung (n [%]) | 6 [28.6 %] | 5 [23.8 %] | |
| Bone and joint (n [%]) | 3 [14.3 %] | 3 [14.3 %] | 0.712 |
| Infectious endocarditis (n [%]) | 2 [9.5 %] | 5 [23.8 %] | |
| Urinary tract (n [%]) | 2 [9.5 %] | 0 [0.0 %] | |
| Meningitis (n [%]) | 0 [0.0 %] | 0 [0.0 %] | |
| Unidentified (n [%]) | 4 [19.0 %] | 2 [9.5 %] | |
| **Quality management parameters** | | | p-value |
| Follow-up blood cultures (n [%]) | 14 [66.7 %] | 19 [90.5 %] | 0.130 |
| Echocardiography (n [%]) | 13 [61.9 %] | 18 [85.7 %] | 0.159 |
| TTE (n [%]) | 6 [28.6 %] | 13 [61.9 %] | 0.062 |
| TEE (n [%]) | 11 [52.4 %] | 13 [61.9 %] | 0.756 |
| Focus/metastasis search (n [%]) | 18 [85.7 %] | 20 [95.2 %] | 0.606 |
| Focus/metastasis control (n [%]) | 6 [28.6 %] | 9 [42.9 %] | 0.520 |
| Adequate antibiotic therapy (n [%]) | 15 [71.4 %] | 17 [81.0 %] | 0.719 |
| AMS consultation (n [%]) | 2 [2 – 3] | 3 [2 – 3] | 0.081 |
| QCI (Median [IQR]) | 14 [66.7 %] | 19 [90.5 %] | 0.130 |
